# A Case of Folliculotropic Mycosis Fungoides Initially Misleading as a Poikilodermatous Rash: The Importance of Gene Rearrangement Testing in Early-Stage Disease (Folliculotropic T-Cell Lymphocytosis)

**DOI:** 10.7759/cureus.98736

**Published:** 2025-12-08

**Authors:** Brian A Moreno, Moises Lutwak, Samantha Sherkin, Francisco Kerdel

**Affiliations:** 1 Dermatology, Lake Erie College of Osteopathic Medicine, Bradenton, USA; 2 Dermatology, Larkin Community Hospital, South Miami, USA; 3 Dermatology, Florida Academic Dermatology Center, Coral Gables, USA

**Keywords:** adult dermatology, case report dermatology, clinical dermatology, complex medical dermatology, dermatology care, dermatology consult, dermatology oncology, general dermatology, medical dermatology, skin disease/dermatology

## Abstract

Folliculotropic mycosis fungoides (FMF) is a distinct and often aggressive variant of cutaneous T-cell lymphoma (CTCL) that can mimic benign dermatoses, particularly in its early stages. We present the case of a 42-year-old man with a chronic, progressively worsening, intensely pruritic, scaly eruption involving the lower back, buttocks, and proximal extremities. Despite initial management with topical corticosteroids, his condition persisted and evolved into poikilodermatous patches with follicular accentuation and hyperpigmented plaques, prompting further evaluation. Serial skin biopsies from the left anterior distal thigh, right posterior shoulder, and right inferior medial lower back revealed superficial and deep perivascular and perifollicular atypical lymphoid infiltrates. Molecular studies demonstrated clonal T-cell receptor (TCR) gene rearrangement, raising concern for early-stage cutaneous T-cell lymphoma with folliculotropic involvement. Given the clinical presentation and histopathologic findings, a diagnosis of early-stage folliculotropic mycosis fungoides was made. The patient was initiated on narrowband ultraviolet B (nbUVB) phototherapy three times per week in addition to topical corticosteroids, with close clinical follow-up.

## Introduction

Mycosis fungoides (MF) is the most common form of cutaneous T-cell lymphoma (CTCL), characterized by an indolent but persistent malignant T-cell proliferation that often mimics benign dermatoses in its early stages. Folliculotropic mycosis fungoides (FMF) is a recognized clinicopathologic variant with a distinct prognosis, defined by preferential infiltration of neoplastic T cells into hair follicles and a tendency to present with follicular papules, plaques, or alopecia rather than classic MF patches. Because FMF frequently exhibits atypical or misleading morphologies, including poikilodermatous changes, early diagnosis can be challenging. This case highlights how the patient’s misleading clinical presentation ultimately required T-cell receptor (TCR) gene rearrangement testing to confirm clonality and establish an early diagnosis of FMF.

The diagnostic workup of early-stage MF can be challenging due to its broad clinical variability and overlapping histopathologic features with benign inflammatory dermatoses. In cases where histology is indeterminate, polymerase chain reaction (PCR)-based TCR clonality assays offer a valuable adjunct for detecting malignant lymphocyte populations. Studies evaluating TCRβ gene rearrangement have demonstrated its utility in confirming clonality in MF, especially when combined with appropriate clinical correlation [1]. Moreover, advanced techniques such as multiplex PCR with heteroduplex analysis and laser-capture microdissection have demonstrated improved sensitivity for early disease detection [2], while GeneScan-based assays provide reproducible profiles that can distinguish reactive from neoplastic infiltrates [3].

Immunophenotyping and TCR gene rearrangement testing together enhance diagnostic accuracy by differentiating cutaneous T-cell lymphomas from pseudolymphomas [4]. Specifically, TCRγ gene analysis and immunohistochemical profiling have been shown to aid in the subclassification of MF and improve the diagnostic yield in subtle or early lesions [5]. Once MF is established, understanding its immunopathogenesis, including the sequential acquisition of genetic and epigenetic abnormalities that lead to clonal expansion of malignant T cells, can inform therapeutic decision-making [6].

While skin-directed therapies remain the cornerstone of early-stage MF management, randomized studies have evaluated low-dose psoralen-UV-A regimens with or without maintenance, demonstrating disease control in a subset of patients [7]. However, certain MF variants, such as FMF, exhibit distinct behavior. FMF is an aggressive subtype characterized by preferential infiltration of neoplastic T cells around hair follicles, resulting in follicular papules, plaques, or alopecia, and is often more resistant to conventional therapies [8]. Clinical recognition is further complicated when FMF mimics atypical morphologies, such as poikiloderma, requiring a high index of suspicion and multiple biopsies.

Accurate diagnosis also hinges on staging and clinical pattern recognition. Guidelines stress that MF may present with a variety of skin changes, including erythematous patches, plaques, and poikilodermatous features, across different stages [9,10]. In poikilodermatous MF, the triad of atrophy, telangiectasias, and mottled pigmentation is often mistaken for benign photodamage or connective tissue disorders, delaying biopsy and targeted testing [11]. Management of MF and its variants typically follows a staged approach integrating skin-directed therapies, biologics, or systemic agents depending on disease severity and subtype [12-14]. For patients with refractory or advanced FMF, novel strategies, such as targeted immune modulation, are currently being investigated to improve long-term outcomes [15].

## Case presentation

**Figure 1:**
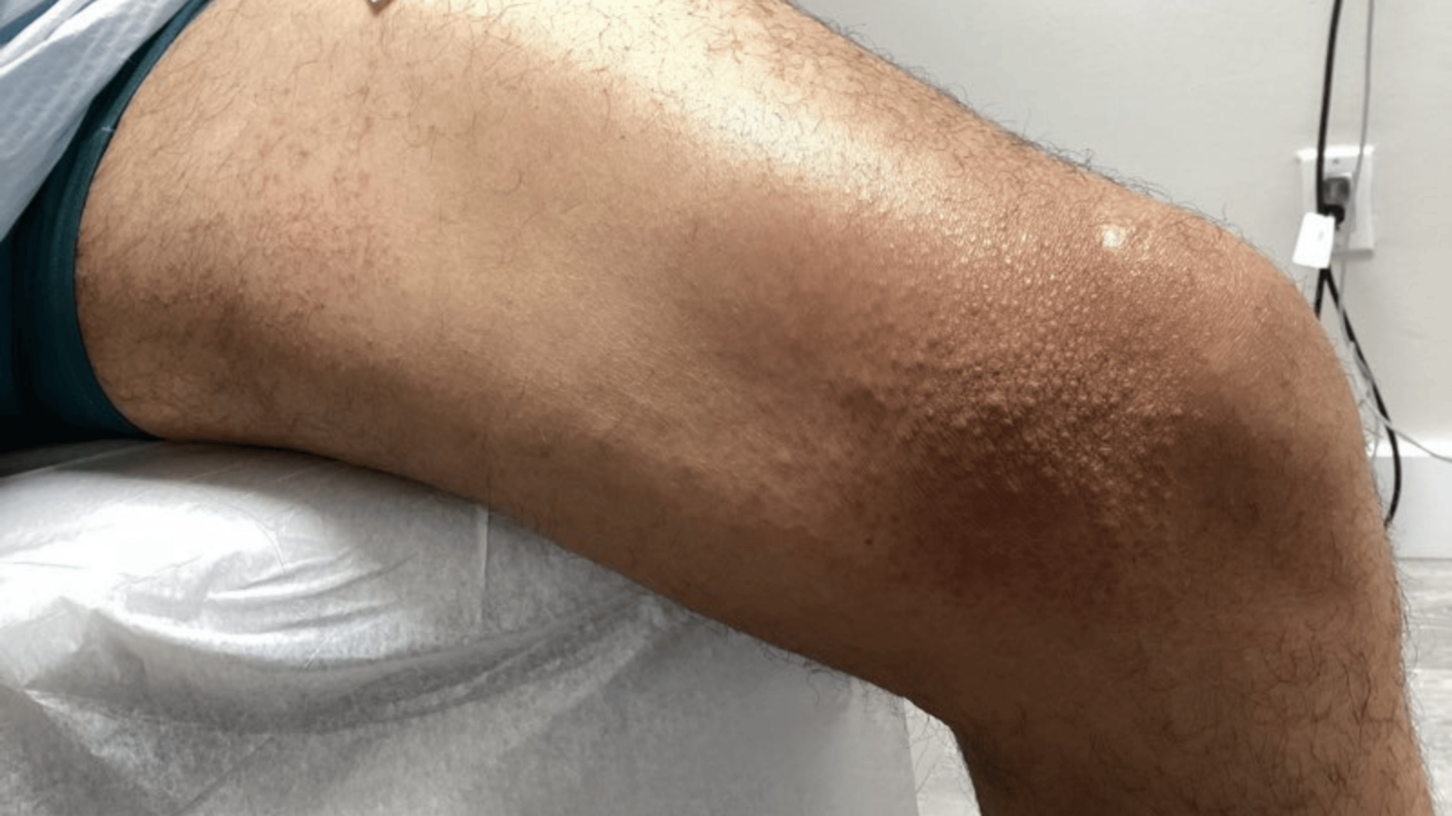
Pruritic, lichenified, hyperpigmented papules and plaques involving the left lower extremity.

**Figure 2:**
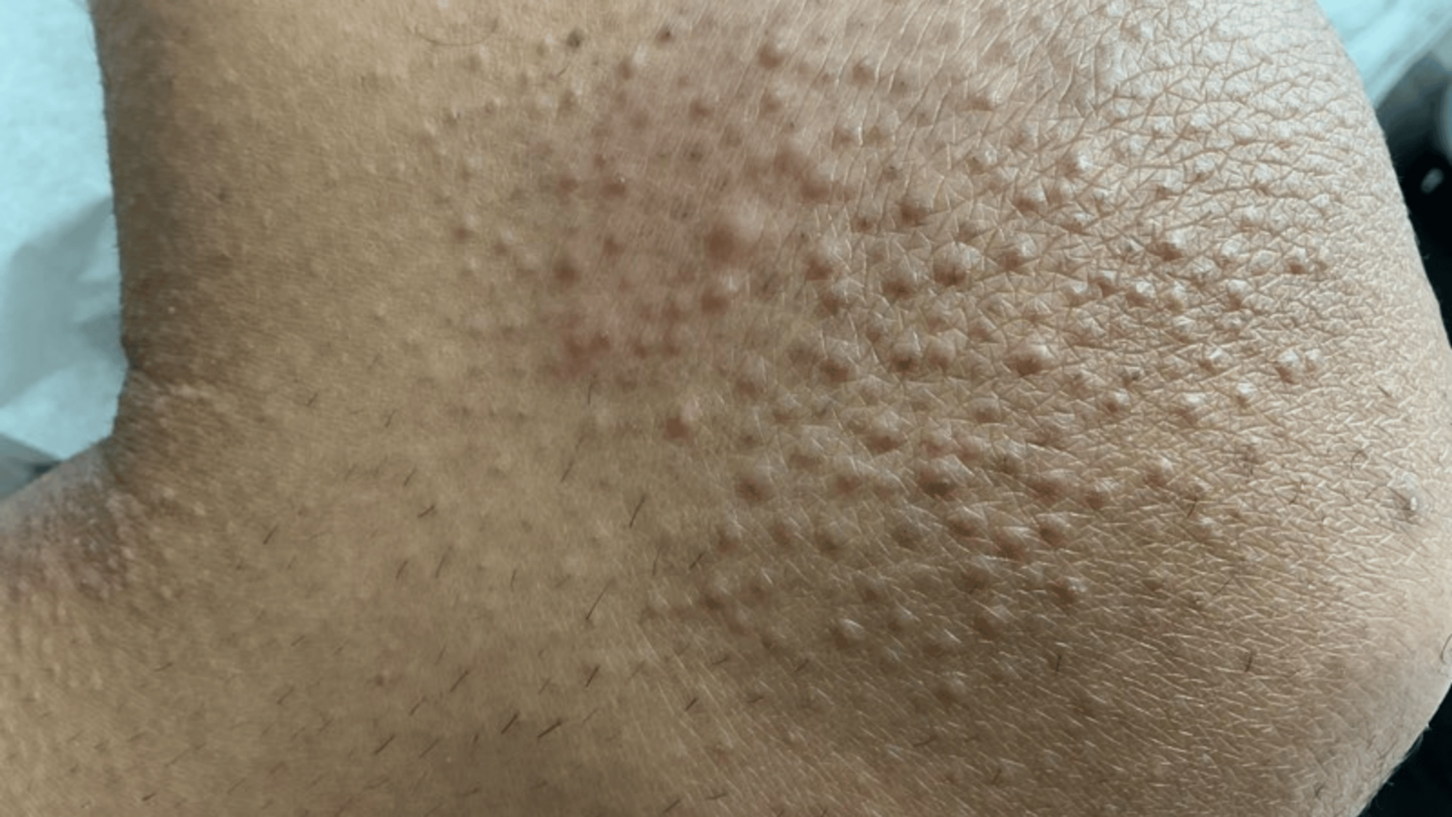
Pruritic, lichenified, hyperpigmented papules and plaques involving the upper extremity.

**Figure 3:**
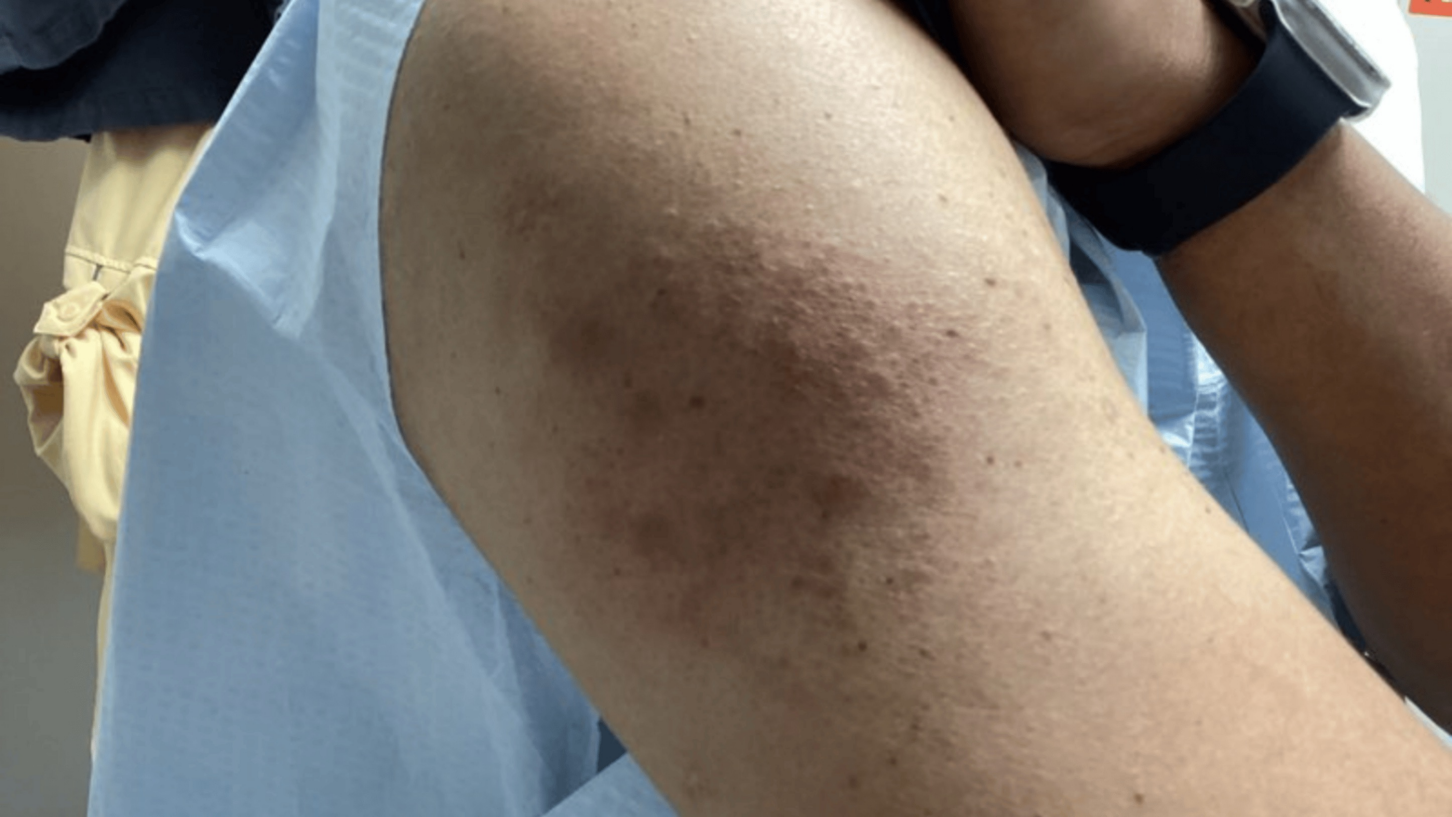
Pruritic, lichenified, hyperpigmented papules and plaques involving the right upper extremity.

**Figure 4:**
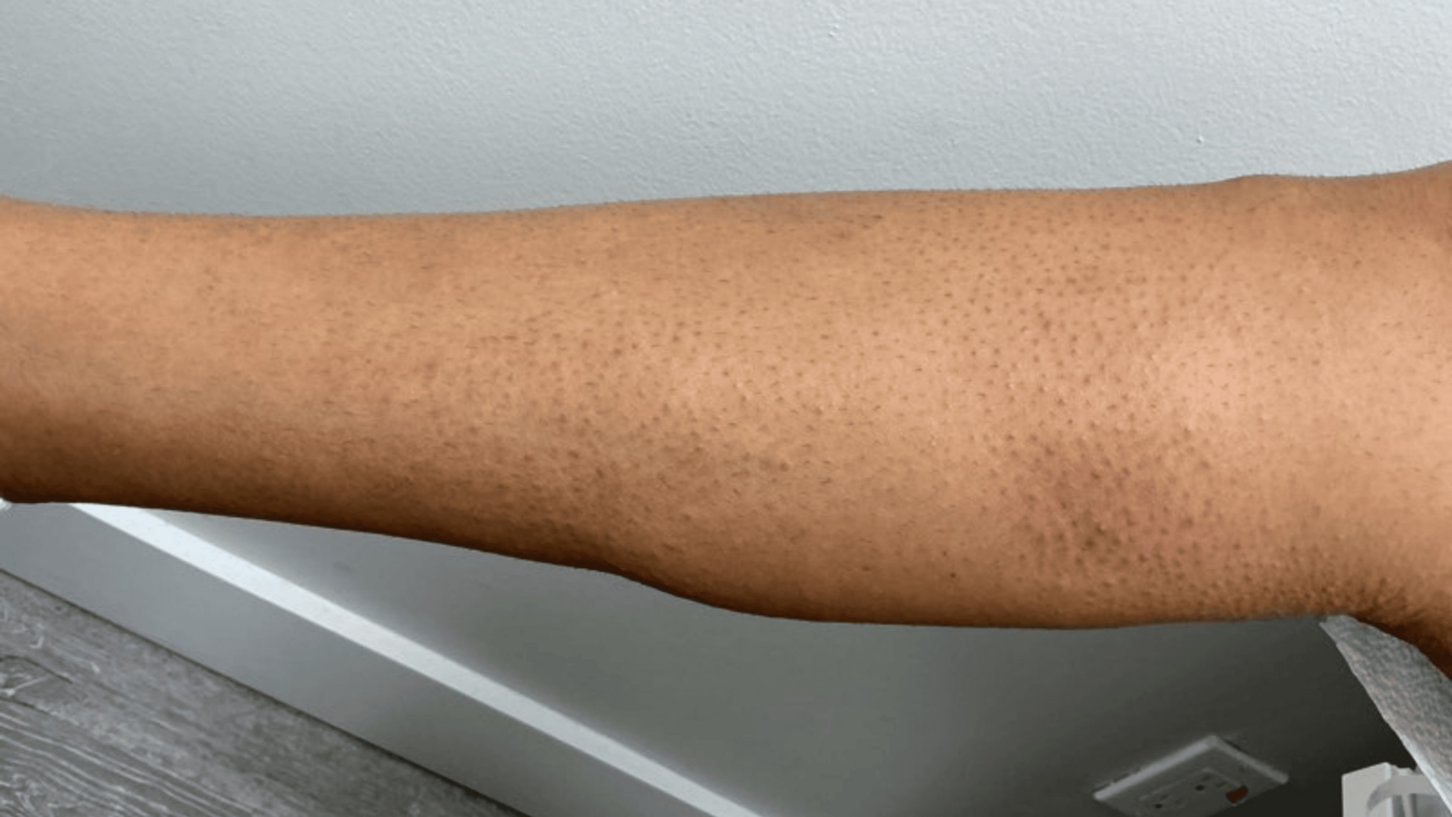
Pruritic, lichenified, hyperpigmented papules and plaques involving the right lower extremity.

A 42-year-old man presented with a chronic history of pruritic, lichenified, hyperpigmented papules and plaques involving the bilateral upper and lower extremities, lower back, buttocks, and portions of the trunk (Figures 1-4). The eruption was intensely pruritic and only mildly responsive to oral antihistamines and topical corticosteroids prescribed by outside providers. Initial clinical impression at our center favored a poikilodermatous or eczematous process, and treatment with triamcinolone 0.1% ointment, hydroxyzine, and continued follow-up was initiated.

**Figure 5:**
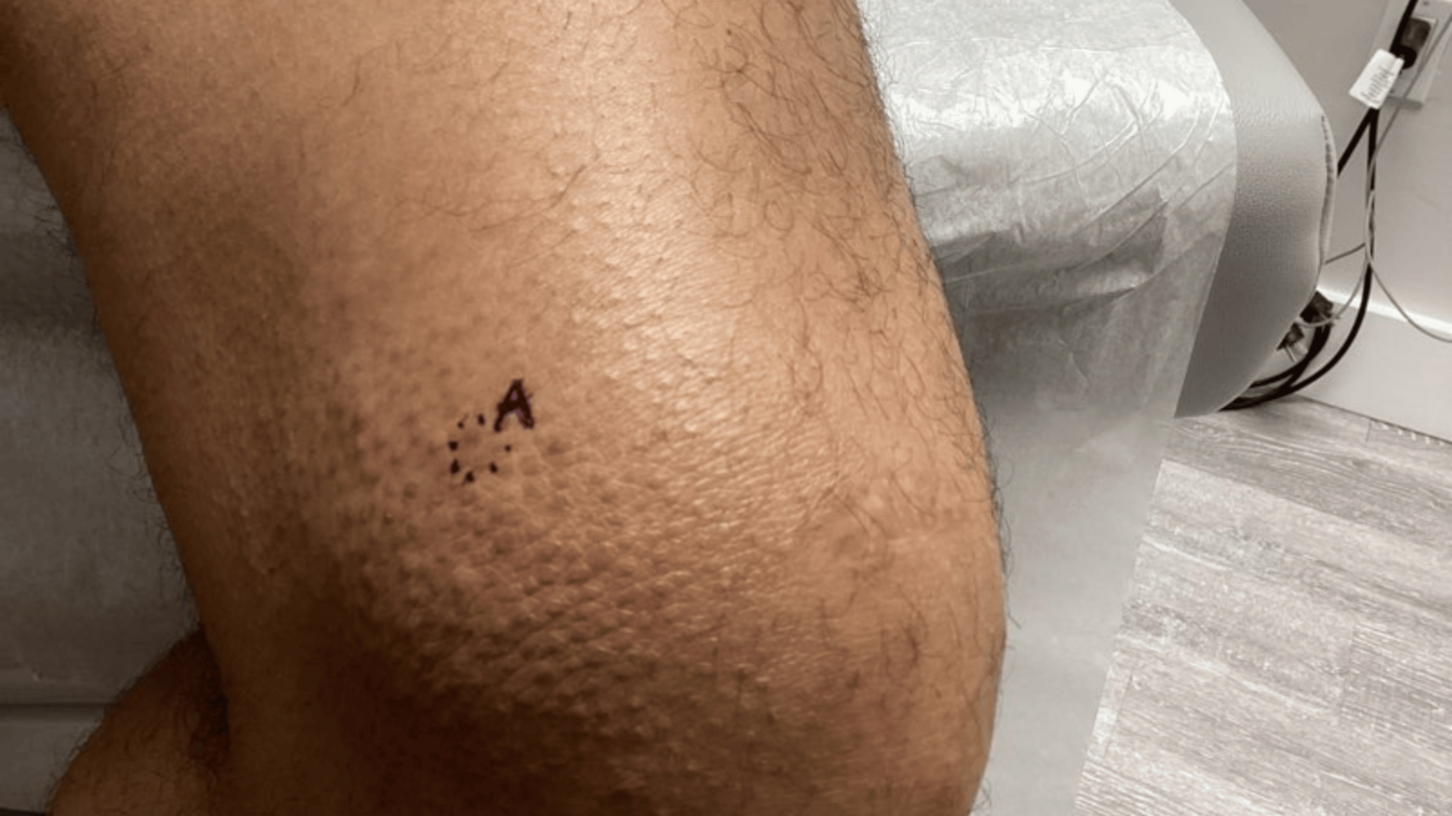
Clinical image demonstrating persistent hyperpigmented papules and plaques with lichenification and poikilodermatous changes on the left anterior distal thigh, prompting punch biopsies due to recalcitrant and evolving distribution.

**Figure 6:**
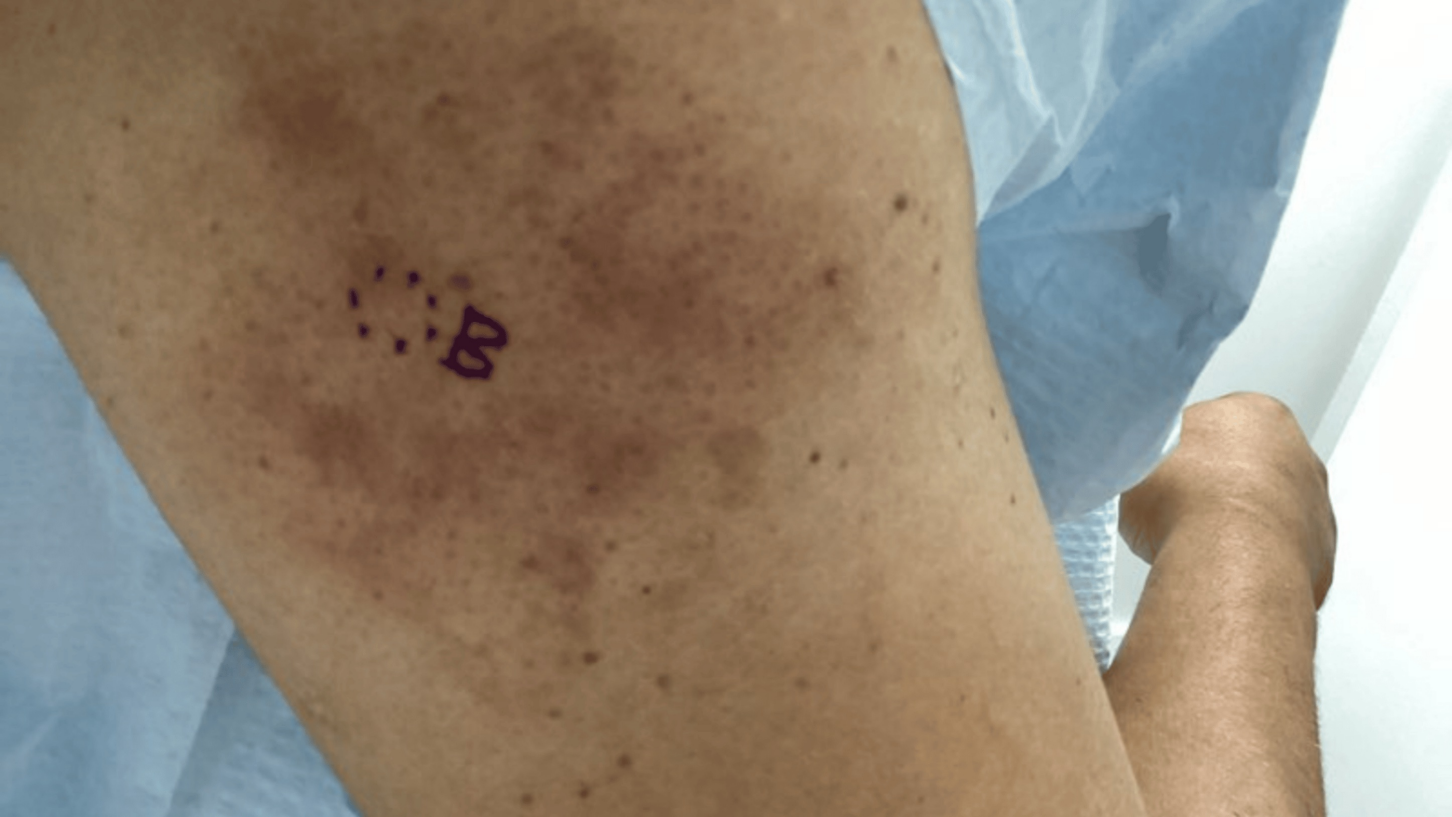
Clinical images demonstrating persistent hyperpigmented papules and plaques with lichenification and poikilodermatous changes on the right posterior shoulder, prompting punch biopsies due to recalcitrant and evolving distribution.

**Figure 7:**
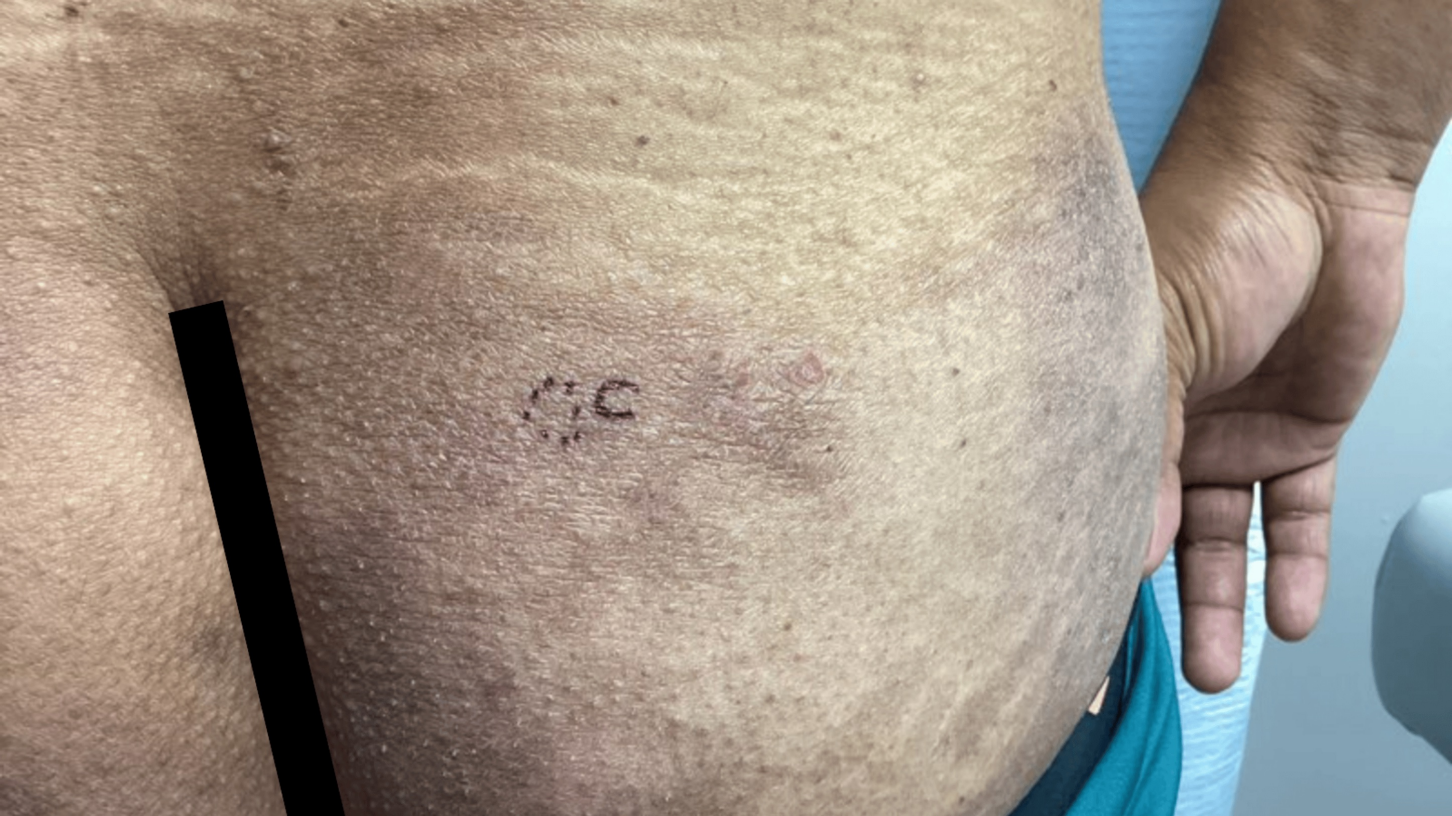
Clinical image demonstrating persistent hyperpigmented papules and plaques with lichenification and poikilodermatous changes on the right inferior medial lower back, prompting punch biopsies due to recalcitrant and evolving distribution.

Over the course of several dermatology visits, the rash persisted despite treatment. On physical examination, there were scattered hyperpigmented papules and plaques with associated lichenification and poikilodermatous features. On repeat examinations, poikilodermatous changes became more apparent, including areas of fine epidermal atrophy, subtle telangiectasias, and mottled hyper- and hypopigmentation. Although classic MF patches were not observed, scattered areas demonstrated follicular accentuation with small, grouped papules, findings that raised suspicion for a folliculotropic variant. Given the recalcitrant nature of the eruption and evolving distribution, a punch biopsy was performed from the left anterior distal thigh, the right posterior shoulder, and the right inferior medial lower back (Figures 5-7).

Histopathology revealed a superficial and deep perivascular and perifollicular atypical lymphocytic infiltrate, raising suspicion for FMF. These findings supported a folliculotropic process, particularly given the presence of atypical lymphocytes surrounding follicular structures rather than remaining limited to the superficial dermis. Although the early biopsies demonstrated features that could overlap with inflammatory dermatoses, the perifollicular pattern and deeper distribution of the infiltrate raised increasing concern for a CTCL. Immunohistochemical staining demonstrated a CD4-predominant T-cell population with loss of CD7 expression. PCR-based T-cell receptor (TCR) gene rearrangement studies detected a clonal T-cell population, confirming the presence of cutaneous T-cell lymphoma with folliculotropic features.

The patient was referred to dermatology-oncology for further staging and management. At follow-up, total body skin examination showed involvement of approximately 25% of the body surface area, with mildly hyperpigmented plaques displaying follicular predominance on the arms, legs, back, and buttocks, and no palpable lymphadenopathy. Baseline laboratory work-up, including complete blood count and general metabolic testing, was ordered prior to initiation of phototherapy. The patient was started on nbUVB phototherapy three times per week and continued topical corticosteroids, with planned serial clinical monitoring.

## Discussion

MF is the most common variant of CTCL and is often misdiagnosed in its early stages due to its resemblance to benign inflammatory dermatoses [1,13]. In this case, the patient presented with features mimicking a poikilodermatous eruption, but histopathologic findings and molecular studies ultimately supported a diagnosis of FMF, a rare and more aggressive subtype [9].

Gene rearrangement studies, such as PCR-based TCR clonality testing, have proven valuable as diagnostic adjuncts when histopathology alone is inconclusive [1-5]. In particular, the detection of a monoclonal T-cell receptor gene rearrangement in our patient strengthened the diagnosis of MF, consistent with prior studies supporting the use of TCR gene rearrangement to distinguish MF from reactive dermatoses [1-3]. The addition of immunophenotyping, as done in this case, further helps to differentiate CTCL from pseudo-T-cell lymphomas and cutaneous pseudolymphomas, which often lack the characteristic CD4+ predominance and CD7 loss seen in MF [4,5].

FMF, the subtype ultimately diagnosed in our patient, displays unique features including folliculotropic infiltrates, perifollicular accentuation, follicular mucinosis, and deeper dermal involvement, often requiring multiple biopsies for detection [9,15]. Unlike classic MF, FMF has a more aggressive clinical course and poorer prognosis, especially when presenting in advanced stages [9,15].

From a therapeutic standpoint, early-stage MF can be managed with skin-directed treatments such as phototherapy. In particular, psoralen plus UVA (PUVA) has demonstrated effectiveness in achieving remission and prolonging disease-free intervals, as shown in a randomized clinical trial evaluating low-dose regimens [7]. In our patient, nbUVB phototherapy was chosen as a practical skin-directed modality to address the 25% BSA involvement and folliculotropic features, with plans for ongoing maintenance depending on clinical response. In contrast, advanced FMF cases may require systemic therapies such as retinoids, interferon, or histone deacetylase inhibitors, though response rates are variable [10-12].

This case emphasizes the importance of combining clinical suspicion, histopathologic analysis, immunophenotyping, and molecular diagnostics to identify MF subtypes accurately. In patients presenting with poikilodermatous or folliculotropic features, early and repeated biopsies alongside clonality testing are essential for establishing diagnosis and guiding management [2,3,6,13].

## Conclusions

This case highlights the diagnostic complexity of early-stage MF, particularly when clinical and histologic features mimic inflammatory dermatoses. The eventual diagnosis of FMF, an aggressive variant of CTCL, was established only after serial biopsies, immunophenotyping, and confirmation of T-cell clonality via gene rearrangement studies. This underscores the critical role of integrating molecular diagnostics with clinical and histopathologic evaluation in cases where suspicion remains high despite initial nondiagnostic results. Prompt and accurate identification of MF subtypes facilitates a timely initiation of targeted therapy, which may improve long-term outcomes. In this case, histopathology revealed atypical perifollicular infiltrates suggestive of FMF, but the findings were not independently definitive. The identification of monoclonal T-cell receptor gene rearrangement provided the crucial diagnostic confirmation of T-cell clonality necessary to establish the diagnosis at an early stage. Continued awareness of MF variants and their protean presentations is essential for dermatologists, pathologists, and oncologists alike.
